# MRT-boost as the last fraction may be the most efficient irradiation schedule for increased survival times in a rat glioma model

**DOI:** 10.1107/S1600577523002606

**Published:** 2023-04-17

**Authors:** Raphael Serduc, Audrey Bouchet

**Affiliations:** a Univ. Grenoble Alpes, INSERM UA7 STROBE, Rue de la Piscine, 38400 Saint-Martin d’Hères, France; b Centre Hospitalier Universitaire Grenoble-Alpes, Maquis du Grésivaudan, 38700 La Tronche, France; c INSERM U1296, Radiation: Defense, Health, Environment, 28 Rue Laennec, 69008 Lyon, France; Australian Synchrotron, Australia

**Keywords:** synchrotron microbeam radiation therapy, radiation boost, brain tumors responses

## Abstract

Synchrotron microbeam radiation therapy (MRT) is based on the spatial fractionation of the incident synchrotron beam into arrays of parallel microbeams, typically a few tens of micrometres wide and depositing several hundred Gray. This high dose, high dose rate, spatially fractionated radiotherapy has a high therapeutic impact on tumors, especially in intracranial locations. MRT leads to better control of incurable high-grade glioma than from homogeneous radiotherapy. The schedule of MRT within a conventional irradiation protocol (three fractions of 11 Gy) of brain tumors was evaluated on the 9L glioma model in rats. MRT delivered as a first fraction increased the median survival time of the animals by four days compared with conventional radiotherapy, while the last MRT fraction improved the lifespan by 148% (+15.5 days compared with conventional radiotherapy, *p* < 0.0001). The most efficient radiation regimen was obtained when the MRT-boost was applied as the last fraction, following two conventional clinical exposures.

## Abstract 2

It is demonstrated that the MRT boost in a 3 × 11 Gy radiation treatment has to be delivered as the last fraction to be more efficient for brain tumor control.

## 1. Introduction

In 1992, Slatkin and colleagues patented a novel form of radiosurgery which stands now on the last step before its clinical transfer (Slatkin *et al.*, 1992 ▸). Microbeam radiation therapy (MRT) uses high-dose-rate synchrotron-generated X-rays spatially fractionated into arrays of parallel microbeams (50 µm wide) delivering hundreds of Grays (Gy) of in-beam radiation dose. Three decades of pre-clinical research have demonstrated the extraordinary radiotolerance of normal brain to microbeam exposures while palliating and even curing aggressive and radioresistant brain tumors [see Engels *et al.* (2020 ▸) and Smilowitz *et al.* (2006 ▸), and, for review, Bouchet *et al.* (2015*a* ▸) and Eling *et al.* (2019 ▸)]. We have previously demonstrated that unidirectional MRT irradiation of rodent gliosarcoma (9L) led to an increase in animal survival compared with unidirectional broad beam (BB) irradiation (Bouchet *et al.*, 2016*a* ▸). MRT and BB were similarly efficient when the MRT valley dose was half that of BB (*i.e.* the dose in between microbeams was two times lower than that administered by BB irradiation) (Bouchet *et al.*, 2016*a* ▸). Unexpected dose biological equivalence (up to 2.5×) compared with conventional radiotherapy (ConvRT) has been reached using a single fraction of multiport MRT (Eling *et al.*, 2021 ▸). Recent data suggested that MRT should be delivered as a boost as part of conventional treatment since exact patient repositioning required for daily delivery of MRT fractions would be impossible because of the submillimetric scale of the microbeams (Potez *et al.*, 2020 ▸). Indeed, MRT could be used at first in patients as a boost or a limited part of a hypofractionated treatment in which larger doses than 2 Gy per fractions are commonly used [*e.g.* brain metastasis, 3 × 11 Gy; glioblastomas boost regimen, 46 + 14 Gy or 50 + 10 Gy (Kazda *et al.*, 2018 ▸)].

The optimal schedule for delivering the MRT-boost has been largely speculated during several radiation-oncology meetings but no experimental data supported any of the evocated assumptions. Here, we determined the optimal radiation regimen for MRT-boost by irradiating, based on a hypofractionated brain metastasis treatment regimen (3 × 11 Gy), on 9L tumor-bearing rats and substituting sequentially one of the three ConvRT fractions by one MRT fraction. Our results highlight that one particular schedule is significantly favorable for improving the MRT-boost efficiency.

## 2. Methods

All operative procedures relating to animal care strictly conformed to the guidelines of the French Government (project authorization numbers 05268.02 and 2017062718191875; authorized lab B3818510002 and B3851610008) and were conducted at European Synchrotron Radiation Facility (ESRF) in France.

The anesthetic procedure used in this study was isoflurane (5% in air) for induction and an intraperitoneal injection of xylazine/ketamine (64.5/5.4 mg kg^−1^) for maintenance during tumor implantation and irradiations.

**Figure 1:**
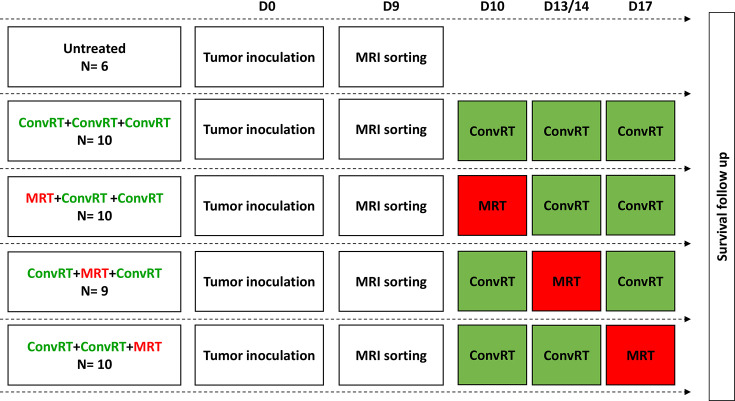
Experimental timeline. Tumor inoculation is considered as day 0 (d0), MRI sorting (d9) and irradiations 10 (d10), 13/14 (d13/14) and 17 (d17) days after tumor inoculation. ConvRT = broad conventional exposures on a Philips X-ray generator (in green); MRT = microbeam radiation therapy (in red). *N* is the number of rats.

The timeline of the experiment is presented in Fig. 1 ▸.

## 3. Tumor implantation and group sorting

Fisher 344 rats (*n* = 45) of 8–10 weeks were implanted with 10^4^ 9L gliosarcoma cells in the right caudate nucleus as described previously (Bouchet *et al.*, 2014 ▸). Briefly, 10^4^ 9LGS cells suspended in a 1 µl DMEM medium were injected into the right caudate nucleus (3.5 mm from the bregma) at a depth of 5.5 mm from the dura. Among rats bearing high-grade orthotopic tumors, animal groups were chosen on the basis of MRI T_2_W [on a Bruker Avance 3 console at 7 T and volume/surface cross coil configuration (Avance III console; Bruker, Germany); ‘Grenoble MRI facility IRMaGE’] in order to distribute the tumors homogeneously size-wise within the following groups of irradiation: **1** – group of untreated animals (*n* = 6); **2** – ConvRT only (three fractions of 11 Gy) (*n* = 10); **3** – ConvRT/ConvRT/MRT: 2 ConvRT fractions of 11 Gy + 1 MRT fraction (*n* = 10); **4** – MRT/ConvRT/ConvRT: 1 fraction of MRT + 2 fractions of 11 Gy ConvRT (*n* = 10); and **5** – ConvRT/MRT/ConvRT: 1 fraction of 11 Gy in ConvRT + 1 fraction of MRT + 1 fraction of 11 Gy in ConvRT (*n* = 9).

## 4. Radiation sources and irradiation schedule

The animals were irradiated every three to four days (due to technical constraints) from day 10 after implantation, either by ConvRT or MRT according to the group sorting.

### 4.1. Conventional exposures on X-ray generator

Anesthetized rats were irradiated individually using orthovoltage photons performed on a Philips X-ray generator operated at 200 kVp, located on the ID17 beamline of the ESRF. Their sagittal suture was positioned on the left side edge of a 1 cm × 1 cm field for dorso-ventral irradiation of the entire rat’s right brain hemisphere (tumor volume/treatment volume ratio from 0.03 to 0.06). A dose rate of ∼1.4 Gy min^−1^ was measured using an ionization chamber (Semiflex 31010, PTW) placed in solid water plates (water equivalent phantom) at 1 cm depth (equivalent to tumor depth) and at room temperature. The ionization chamber and solid water plates were fixed at the same position and distance from the irradiation source as the rat head. A dose of 11 Gy was delivered to treated rats from day 10 after tumor implantation, depending on the animal group.

### 4.2. MRT exposures

Irradiations were performed at the ID17 biomedical beamline at the European Synchrotron Radiation Facility (Grenoble, France). The wiggler produces a wide spectrum of photons (50 to 350 keV; median energy: 90 keV) at a dose rate of ∼16000 Gy s^−1^. The beam was shaped into an array of thin, quasi-parallel microbeams using a multislit collimator (Bräuer-Krisch *et al.*, 2010 ▸). The doses were calculated by means of the Monte Carlo method [detailed dosimetry protocols by Martínez-Rovira *et al.* (2012 ▸)]. Fifty microbeams, 50 µm thick, spaced 400 µm apart, were used (8 mm × 12 mm irradiation field). Prone rats were irradiated using two orthogonal (lateral and anteroposterior) and coplanar ports at 126 Gy in-microbeam dose, which resulted in a 5.5 Gy valley dose equivalent to that administered by the 11 Gy ConvRT fractions.

## 5. Treatments evaluation

The survival curves were established using six rats for control, ten for ConvRT, ten for MRT/ConvRT/ConvRT, nine for ConvRT/MRT/ConvRT and ten for ConvRT/ConvRT/MRT groups. The time between the first fraction irradiation and death was recorded as the survival time; one day was added in cases of euthanasia. Kaplan Meier survival data and median survival time (MST) were compared using a log rank test in *GraphPad Prism* version 9.0.1 (GraphPad Software, La Jolla, California, USA; https://www.graphpad.com/).

The average increase in life span (% ILS) was calculated using the following formula: % ILS = (*T*/*C* × 100) – 100, where *T* and *C* are the mean survival days of treated and control groups of rats, respectively.

## 6. Results

### 6.1. MRT improves tumor-bearing rat’s lifespan whatever the schedule used compared with ConvRT only

**Figure 2:**
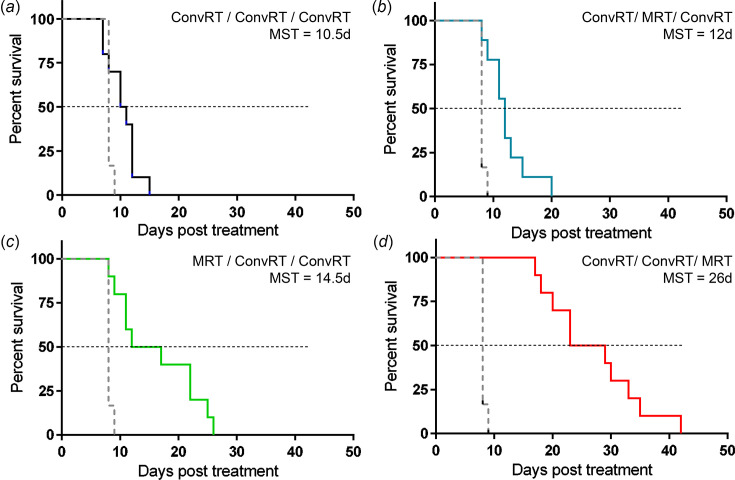
MRT-boost improves survival of 9L tumor-bearing rats. Kaplan Meier survival curves of untreated (CTRL, dash gray) and treated by (*a*) ConvRT/ConvRT/ConvRT (solid black), (*b*) ConvRT/MRT/ConvRT (solid blue), (*c*) MRT/ConvRT/ConvRT (solid green) and (*d*) ConvRT/ConvRT/MRT (solid red).

Kaplan Meier survival curves are shown in Fig. 2 ▸. MST of the control group reached 8 days after tumor sham irradiation. Three fractions of 11 Gy delivered by ConvRT alone increased significantly the lifespan of the animals by 2.5 days (*p* = 0.034, Table 1 ▸) compared with controls. Whatever the position during the treatment, MRT-boost improved MST of 9L-bearing rats compared with the control and the conventionally irradiated groups. Indeed, survival medians of the animals were increased by 4, 6.5 and 18 days (compared with controls) when MRT was delivered during, before and after, respectively, the conventional treatment. When compared with the latter, MRT survival curves were significantly different when the microbeam fraction was delivered as a first or a last fraction (*p* = 0.028 and *p* < 0.001, respectively).

### 6.2. Microbeams delivered as the last fraction is the most efficient schedule for MRT-boost in a rat glioma model

**Figure 3:**
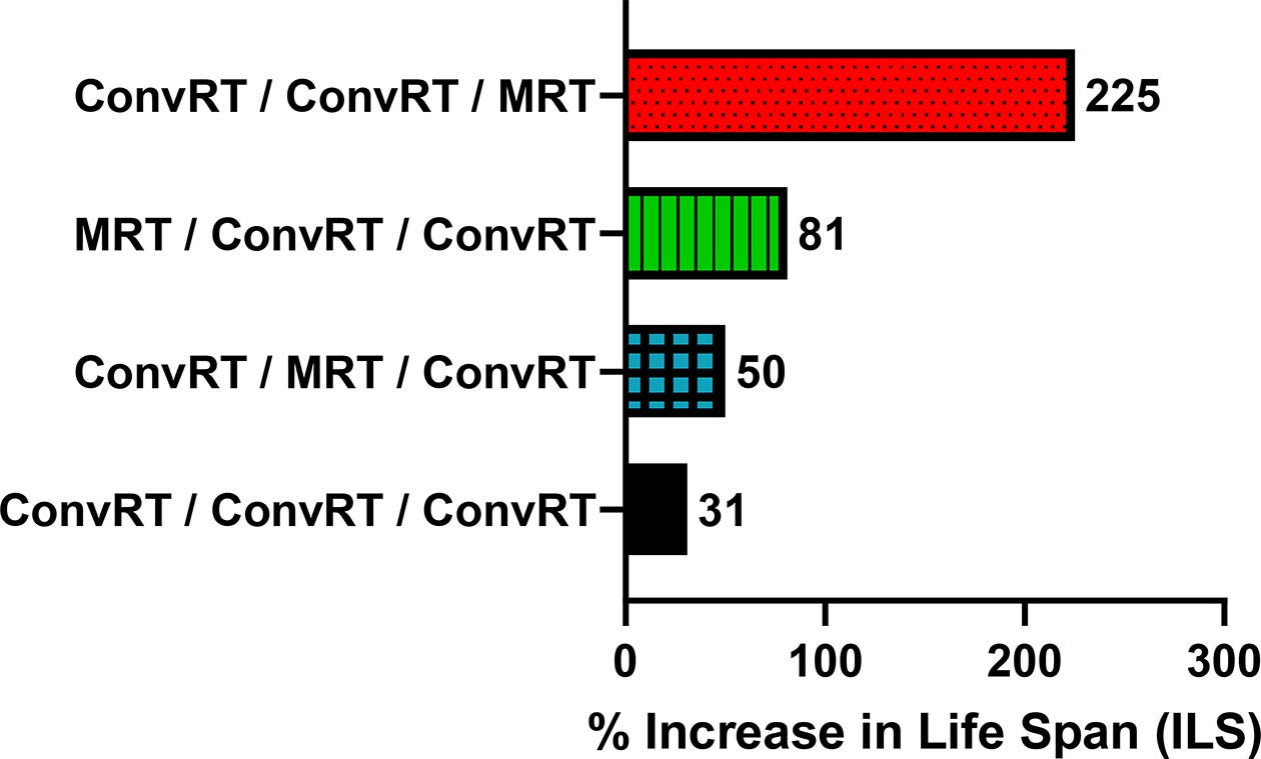
Percentage of average increase in life span (%ILS) obtained with different schedules of irradiation compared with none irradiated animals group. %ILS of rats treated by ConvRT/ConvRT/ConvRT (black): 31%; ConvRT/MRT/ConvRT (blue): 50%; MRT/ConvRT/ConvRT (green): 81%; or ConvRT/ConvRT/MRT (red): 225%.

Considering MRT fractions only, it appeared that the lowest efficiency was obtained when the boost was applied in between two ConvRT fractions. Indeed, MRT delivered at the beginning or at the end of the irradiation schedule allows improvements in survival times. MRT delivered as a first fraction increased MST of the animals by 4 days compared with ConvRT, while the last MRT fraction improved the lifespan by 148% (+15.5 days compared with ConvRT, *p* < 0.0001). MRT-boost as the last fraction appeared as the most efficient irradiation schedule used in this study (0.0061 < *p* < 0.0001, see Table 1 ▸). The scheme with ConvRT irradiations only increased the ILS by 31% compared with untreated animals. MRT delivered as a first, intermediate or last fraction resulted in an ILS of 81, 50 and 225%, respectively (Fig. 3 ▸).

## 7. Discussion

The optimal schedule for delivering the MRT-boost has been largely debated over recent years, notably during meetings organized by the COST action ‘Innovative Methods in Radiotherapy and Radiosurgery using Synchrotron Radiation (SYRA3)’. Fractions of MRT applied after several clinical irradiations (ConvRT) led to an increase in lifespan of glioblastoma-bearing rats (F98 tumor) compared with a total treatment with clinical irradiation (Potez *et al.*, 2020 ▸), but the most efficient time to deliver MRT within a time-fractionated hospital protocol remained to be properly defined. Our results, obtained on gliosarcoma-bearing rats, highlighted that MRT substituted in one of the three fractions of ConvRT (3 × 11 Gy), whatever the schedule of the substitution, increases the radiotherapeutic efficiency. However, the use of MRT as the last fraction of the treatment sequence provides the best survival rate (Fig. 2 ▸), with a significant increase in median survival of 3.25 times compared with untreated (*p* < 0.0001, Table 1 ▸) *versus* only 1.3 times for ConvRT without MRT.

Schültke *et al.* (2022 ▸) stated that a high dose rate boost must come first to improve the overall treatment efficiency. However, the conclusions of their study are based on *in vitro* experiments while *in vivo* preclinical data have already shown that an MRT boost could be significantly more efficient than conventional treatments (Bouchet *et al.*, 2016*b* ▸), and we demonstrate in the present study that an MRT-boost is significantly more efficient as the last fraction. In previous work, Potez and colleagues have shown that two MRT-boost fractions, after three BB irradiations, stop F98 tumor growth and increase the lifespan of F98-bearing intracranial rats more than five BB irradiation fractions (Potez *et al.*, 2020 ▸). Here, we confirm the potential of MRT as a boost in substitution of a conventional fraction in a multi-fractionated regimen on another high-grade brain tumor model (9L tumor) (Fig. 2 ▸). The scheme of irradiation with the MRT fraction delivered in between two ConvRT fractions is the least efficient since it led to the lowest MRT’s MST (Fig. 2 ▸, Table 1 ▸). MRT used as a final fraction increased the lifespan by 225% compared with the untreated group and by 148% compared with multi-fractionated conventional irradiation (Fig. 3 ▸).

Several assumptions, which obviously need to be verified *in vivo*, could explain these results. MRT reduces the 9L blood volume and induces tumor hypoxia (Bouchet *et al.*, 2010 ▸, 2013*a* ▸), and, when prescribed at the start of treatment, the MRT-boost might reduce the radiotherapeutic efficiency of the following ConvRT fractions. These additional conventional fractions may also decrease the anti-tumor inflammatory response observed after microbeam exposures (Bouchet *et al.*, 2013*b* ▸, 2016*a* ▸; Eling *et al.*, 2021 ▸; Potez *et al.*, 2018 ▸; Ibahim *et al.*, 2016 ▸; Yang *et al.*, 2019 ▸; Brönnimann *et al.*, 2016 ▸). Several biological mechanisms underlying tumor response to microbeams (Bouchet *et al.*, 2013*b* ▸, 2016*a* ▸; Eling *et al.*, 2021 ▸; Potez *et al.*, 2018 ▸; Ibahim *et al.*, 2016 ▸; Yang *et al.*, 2019 ▸; Brönnimann *et al.*, 2016 ▸) can be transposed to this boost regimen and delineate MRT action when delivered as a last fraction. MRT might (i) damage the remaining vascular entities, even in a hypo-vascularized tumor model (Bouchet *et al.*, 2017 ▸; Potez *et al.*, 2019 ▸), (ii) destroy radioresistant tumor cells which survived to 2 × 11 Gy (Barth, 1998 ▸) and (iii) stimulate macrophage invasion which would sustain and prolong anti-tumor mechanisms (Eling *et al.*, 2021 ▸; Bouchet *et al.*, 2013*b* ▸, 2015*b* ▸).

After 30 years of multidisciplinary research and preclinical studies, the transfer of synchrotron microbeam radiation therapy is now feasible and expected before 2026 for patients with glioblastoma. The final translational step is under study since a veterinary clinical trial started two years ago on glioma-bearing dogs. This Phase I trial aims at evaluating MRT (single fraction) induced side effects and neurotoxicity. It will be followed by an MRT-boost trial to demonstrate MRT relevance in glioblastoma management. In early 2021, the Grenoble University Hospital gathered a ‘local task force’ which launched the procedures necessary for the first human clinical trial of MRT for glioblastomas. The benefit of dose escalation in the treatment of glioblastoma remains controversial but recent meta-analysis highlights a survival gain in patients (Singh *et al.*, 2021 ▸). Marchionni *et al.* (2020 ▸) improved overall survival by 11 months by delivering a 20 Gy boost (four daily × 5 Gy) to glioblastoma patients (*p* = 0.004). Five daily fractions of 5 Gy of the 67% isodose (maximum dose in the planning target volume of 37.5 Gy) yielded a significantly longer progression free survival for recurrent glioblastomas (from four months without boost to nine months with boost) (Arpa *et al.*, 2020 ▸). MRT would be relevant and efficient for patients for whom a boost or a limited part of a hypo-fractionated treatment is required to increase tumor control or slow recurrence (Kazda *et al.*, 2018 ▸). Due to its unique submillimetric irradiation geometry, MRT cannot be daily administrated using the same trajectories and requires multiple entry ports to deliver the total prescribed radiation to the lesion. The advantage of temporal fractionation of MRT ports, as technically shown by Fernandez-Palomo *et al.* (2020 ▸) and Serduc *et al.* (2009 ▸), needs to be questioned but the use of multiports for boost delivery is undisputable since Eling *et al.* (2021 ▸) demonstrated that tumor control improves exponentially with increasing number of ports. One unique boost fraction of 10 Gy valley maximum delivered *via* five ports might reach biological equivalent doses up to 25 Gy (Eling *et al.*, 2021 ▸) which makes the MRT-boost particularly relevant for such aggressive gliomas.

To conclude, this straightforward study demonstrates that MRT-boost improves the overall efficacy of ConvRT at controlling malignant gliomas and should be prescribed as a sequential boost following conventional fractions. To improve the MRT boost efficiency, we recommend to deliver the total radiation dose through a maximum number of ports, and the relevance of temporal fractionation in between ports needs to be further studied and demonstrated.

## Figures and Tables

**Table 1:** Log-rank test comparison between groups

	Survival log-rank tests comparisons (*p* value)					
	MST	Ctrl	BB/BB/BB	MRT/BB/BB	BB/MRT/BB	BB/BB/MRT
Ctrl	8	x	0.034	0.0007	0.013	<10^−4^
BB/BB/BB	10.5		x	0.027	0.234	<10^−4^
MRT/BB/BB	14.5			x	0.102	0.006
BB/MRT/BB	12				x	<10^−4^
BB/BB/MRT	26					x
